# Evaluation of applying IHC4 as a prognostic model in the translational study of Intergroup Exemestane Study (IES): PathIES

**DOI:** 10.1007/s10549-017-4543-7

**Published:** 2017-11-24

**Authors:** M. C. U. Cheang, J. M. Bliss, G. Viale, V. Speirs, C. Palmieri, A. Shaaban, P. E. Lønning, J. Morden, N. Porta, J. Jassem, C. J. van De Velde, B. B. Rasmussen, D. Verhoeven, J. M. S. Bartlett, R. C. Coombes, On Behalf of the PathIES Sub-Committee

**Affiliations:** 1 The Institute of Cancer Research, Clinical Trials and Statistics Unit (ICR-CTSU) Section of Clinical Trials, Sir Richard Doll Building, Sutton, SM2 5NG UK; 2 Department of Pathology, European Institute of Oncology, Via Ripamonti 435, 20141 Milan, Italy; 3 Leeds Institute of Molecular Medicine, University of Leeds, St James’s University Hospital, Wellcome Trust Brenner Building, Leeds, LS9 7TF UK; 4 Department of Molecular and Clinical Cancer Medicine, University of Liverpool, Liverpool, L69 3BX UK; 5 Department of Pathology, Queen Elizabeth Medical Centre, Queen Elizabeth Hospital, University Hospitals Birmingham NHS Foundation Trust and the University of Birmingham, Birmingham, UK; 6 Department of Oncology, University of Bergen, Haukeland University Hospital, 5021 Bergen, Norway; 7 Department of Oncology and Radiotherapy, Medical University of Gdansk, 7 Debinki St, 80-211 Gdansk, Poland; 8 Department of Surgery, Leiden University Medical Center, Albinusdreef 2, 2300 ZA Leiden, Netherlands; 9 Department of Pathology, Herlev and Gentofte Hospital, Copenhagen, Denmark; 10 Department of Medical Oncology, AZ Klina, Braschaat, Belgium; 11 Transformative Pathology, Ontario Institute for Cancer Research, MaRS Centre, 661 University Avenue, Suite 510, Toronto, ON M5G 0A3 Canada; 12 Department of Cancer and Surgery, Faculty of Medicine, Imperial College London, Du Cane Road, London, W12 0NN UK

**Keywords:** Breast cancer, Aromatase, Prognosis

## Abstract

**Background:**

Intergroup Exemestane Study (IES) was a randomised study that showed a survival benefit of switching adjuvant endocrine therapy after 2–3 years from tamoxifen to exemestane. This PathIES aimed to assess the role of immunohistochemical (IHC)4 score in determining the relative sensitivity to either tamoxifen or sequential treatment with tamoxifen and exemestane.

**Patients and methods:**

Primary tumour samples were available for 1274 patients (27% of IES population). Only patients for whom the IHC4 score could be calculated (based on oestrogen receptor, progesterone receptor, HER2 and Ki67) were included in this analysis (*N* = 430 patients). The clinical score (C) was based on age, grade, tumour size and nodal status. The association of clinicopathological parameters, IHC4(+C) scores and treatment effect with time to distant recurrence-free survival (TTDR) was assessed in univariable and multivariable Cox regression analyses. A modified clinical score (PathIEscore) (*N* = 350) was also estimated.

**Results:**

Our results confirm the prognostic importance of the original IHC4, alone and in conjunction with clinical scores, but no significant difference with treatment effects was observed. The combined IHC4 + Clinical PathIES score was prognostic for TTDR (*P* < 0.001) with a hazard ratio (HR) of 5.54 (95% CI 1.29–23.70) for a change from 1st quartile (Q1) to Q1–Q3 and HR of 15.54 (95% CI 3.70–65.24) for a change from Q1 to Q4.

**Conclusion:**

In the PathIES population, the IHC4 score is useful in predicting long-term relapse in patients who remain disease-free after 2–3 years. This is a first trial to suggest the extending use of IHC4+C score for prognostic indication for patients who have switched endocrine therapies at 2–3 years and who remain disease-free after 2–3 years.

**Electronic supplementary material:**

The online version of this article (10.1007/s10549-017-4543-7) contains supplementary material, which is available to authorized users.

## Introduction

The use of aromatase inhibitors (AIs) within the adjuvant setting, either upfront or sequentially before or after tamoxifen, has now been established given the results of several international studies [1–7]. Recently Goss et al. reported results of MA.17R that there was a reduction in contralateral breast cancers and increased disease-free survival, even though there was no overall survival benefit, supporting the extended use of an AI as adjuvant endocrine therapy for 10 years [8]. However, there is still considerable uncertainty as to whether such treatment is necessary for all patients and whether some patients can be treated solely with either tamoxifen or AI alone or switched to AI following tamoxifen treatment such as was done in the IES trial [3]. The IES (Intergroup Exemestane Study) trial continues to report, even in its final analysis, at a median follow-up time of 12 years, a modest improvement in overall survival for those who ‘switched’ treatments (in preparation).

Whilst women with hormone receptor positive breast cancer can acquire resistance to endocrine treatment, it currently remains uncertain how this resistance occurs, and whether different mechanisms of resistance between the two treatment types exist. Several gene expression assays have been developed with the aim of distinguishing those patients who will relapse early on adjuvant endocrine therapy, including the Prosigna^®^, Oncotype DX^®^, EndoPredict^®^ and Breast cancer Index™ but, as yet, none have been evaluated for their capacity to distinguish benefit from different forms of endocrine therapy.

We had established a translational group (PathIES) as part of the IES trial to evaluate the potential role of various candidate biomarkers to distinguish the effectiveness of tamoxifen and AI. This group has already reported the results of ERβ variants and its possible role in helping to predict appropriate endocrine therapy for patients in IES [9].

The immunohistochemical (IHC) 4 + Clinical (C) score is a prognostic tool based on quantitative assessment of immunohistochemical biomarkers (ER, Progesterone receptor (PgR), HER2 and Ki67) and the clinicopathologic variables (tumour grade, size, nodal status, tumour grade, treatment with AI or tamoxifen) [10–12]. The IHC4+C was developed to predict the residual risk of distant recurrence at 9 years in postmenopausal women with ER positive tumours treated with 5 years of adjuvant endocrine therapy only (i.e. no chemotherapy) [13, 14]. However, to date, there is no report to evaluate the prognostic value of IHC4+C to patients who have received adjuvant tamoxifen for 2–3 years, followed by subsequent exemestane treatment to complete a total of 5 years endocrine therapy.

We consider it important to examine the role of the IHC4 score in predicting prognosis in patients who are switched from tamoxifen to an AI since this would provide a different cohort from other studies given that we include only those who remain disease-free at 2–3 years. This cohort therefore excludes patients who relapse early but more closely resembles the cohort in whom continuation of therapy beyond 5 years will increasingly be considered. We also report here on the role of IHC4 score in determining the relative sensitivity to either tamoxifen or sequential treatment with tamoxifen and exemestane.

## Patients and methods

### Study design

IES was a multicentre, international, randomised, double-blind phase III study comparing exemestane 25 mg/day to tamoxifen 20 mg/day (30 mg in Denmark) for 2–3 years in postmenopausal women with ER+/unknown primary breast cancer who remained disease-free after receiving adjuvant tamoxifen therapy for 2–3 years. The study recruited 4724 postmenopausal women from 37 countries (366 centres) between 1998 and 2003 and has shown a survival benefit for those with ER+/unknown cancers from switching to exemestane after 2–3 years tamoxifen [15, 16]. PathIES is a retrospective translational study that aims to identify markers predictive of response or resistance to tamoxifen or an AI. Pathological samples from the primary surgery (at least 2 years before randomisation into the main IES trial i.e. between 1996 and 2001) were collected retrospectively from 1274 women enrolled in the IES (27% of IES patients). This article presents results from the analysis of IHC4 (ER, PgR, HER2 and Ki67) and IHC+C (IHC4 + clinical score based on nodal status, pathological tumour size, grade and age) on PathIES participants. Only PathIES participants for whom IHC4 could be calculated were included in this analysis. All clinical data used in the analyses were based on the snapshot taken for the most recent IES publication (median follow-up time was 91 months) [16] and the REMARK [17] criteria were employed for data reporting.

### Patients

Patients were eligible for enrolment in the IES study if they had histologically confirmed, completely resected unilateral invasive breast carcinoma positive for ERα or that was of unknown receptor status. Patients were postmenopausal and had received adjuvant tamoxifen therapy for at least two years but no more than three years and one month. The study design, detailed eligibility criteria and treatment schedules have been previously described [3]. Formalin-fixed paraffin-embedded (FFPE) tumour samples were retrospectively collected in accordance with institutional guidelines, ethics requirements and national laws. Laws and regulations at the time of tissue collection on consent requirements, collection of archived FFPE samples from patients that were deceased and international sample transfers limited the number of countries that could participate in PathIES.

### Immunohistochemistry

FFPE tissue blocks were received at the central laboratory, and tissue microarrays (TMAs) were constructed as described [9], except where lesions were of insufficient size. Pathology laboratories that were unable to submit FFPE tumour blocks were requested to provide 4–5 micron whole sections. Full information on immunohistochemistry was previously described [9].

## Statistical analyses

The primary endpoint for this study was time to distant recurrence (TTDR) defined as time from random assignment to treatments to distant recurrence, death from breast cancer or unknown cause without prior recurrence. The clinicopathological characteristics of patients selected for this analysis to those not selected (due to unavailable tissue for the analysis, or unavailable markers for IHC4 assessment) were tabulated. No allowance has been made for multiple testing.

### Calculation of IHC4 score and evaluation of its prognostic value among PathIES participants

Analysis was limited to ER+ PathIES patients as assessed centrally by either ≥ 1% positive stained cells or H-score ≥ 1 or Allred ≥ 3. The Cuzick et al. [10] algorithm was adopted as follows, to derive the IHC4 score, which in combination with a clinical score (nodes, grade, age, tumour size) was tested for its prognostic value on our data.

$$ {\text{IHC4}}\;{ = }\; 9 4. 7x\;\;[ - 0.1\;{\text{ER}}_{10} - 0.079\;{\text{PgR}}_{10} \; + \;\;0.586\;{\text{HER2 + 0}} . 2 4 0 { }x\;\ln (1\; + \;4\;x\;{\text{ki67}}) \, ] \, $$

$$ \begin{aligned} {\text{Clinical score = 100 }}x\;[0.417\;N_{1 - 3} + \;1.566\;N > 3\; + \;\;0.93\;x\;(0.497\;T_{1 - 2} + \;0.882\;T_{2 - 3} \hfill \\ + 1.838\;T > 3\; + \;0.559\;Gr_{2} + 0.970\;Gr_{3} \; + \;0.130\;{\text{Age}}\;{ > }\; 6 5 ]\hfill \\ \end{aligned}. $$

In brief, the ER [10] was equivalent to ER H-score divided by 30 and PgR [10] equivalent to PR percentage of positive tumour nuclei cells divided by 10. The range of ER10 and PgR would be 0–10. HER2 was considered positive if IHC staining was 3+ and negative for IHC 0, 1+, 2+. Ki67 score was transformed as ln(1 + (4 × ki67)). The IHC4 risk groups were categorised as follows: quartile (Q) 1: < 25%, Q2–Q3: ≥ 25% and < 75%, Q4: ≥ 75%).

For the clinical score,* N*_j_,* T*_j_,* G*_rj_ and Age_j_ denote categories of nodal status (N0, 1–3 N+ , > 3 N+), tumour size (< 1 cm (*T*_0_), 1–2 cm (*T*_1–2_), 2–3 cm (*T*_2–3_) and > 3 cm (*T* _>3_)), grade (I, II, III) and age (< 65, ≥ 65).

The anastrozole versus tamoxifen effect term was deemed inappropriate for the exemestane effect on the PathIES data to validate the prognostic model of IHC4+C and therefore it was omitted. The IHC4+C risk groups were also categorised based on the quartiles (Q) (Q1: < 25% vs Q2–Q3: ≥ 25% and < 75% vs. Q4: ≥ 75%).

Kaplan–Meier plots, log-rank tests and Cox proportional hazards models, as appropriate, were used to compare how time to distant recurrence varied according to the IHC4 and IHC4+C risk groups. The significance of treatment effect with risk groups was determined by an interaction test in the multivariable Cox model.

A calibration plot comparing the predicted and observed probability of distant relapse by 10 years assessed the performance of the IHC4+C prognostic score. Patients were divided into ten groups according to their 10th percentiles of IHC4+C score; mean predicted values within each group were compared to the observed Kaplan–Meier estimates obtained for each group at 10 years.

### Adjusting the prognostic clinical score model using PathIES parameters

To retain the comparability with the original IHC4+C model as reported by Cuzick et al., we used the same criteria to categorise the following variables: age, nodal status, tumour size and grade. The association of clinicopathological parameters, IHC4 score (included as continuous variable) and the PathIES treatment effect (tam→exem vs. tam alone) with survival data was assessed in a univariable Cox regression analysis. For the multivariable Cox regression analysis, we applied a stepwise backward strategy to select the most prognostic factors, whilst forcing the selection to keep treatment in the model, as assessed by a significance level of 10% if not lower.

## Results

### Characteristics of PathIES participants

Of the 4724 postmenopausal women with ER+/unknown primary breast cancer included in the IES trial, 1483 from 89 centres were recruited into PathIES study. Of those, material was available for 1274 women, 27% of the IES population or 86% of the population recruited from centres participating in PathIES (Supplementary Table 1).

### IHC4 scores

**Fig. 1:**
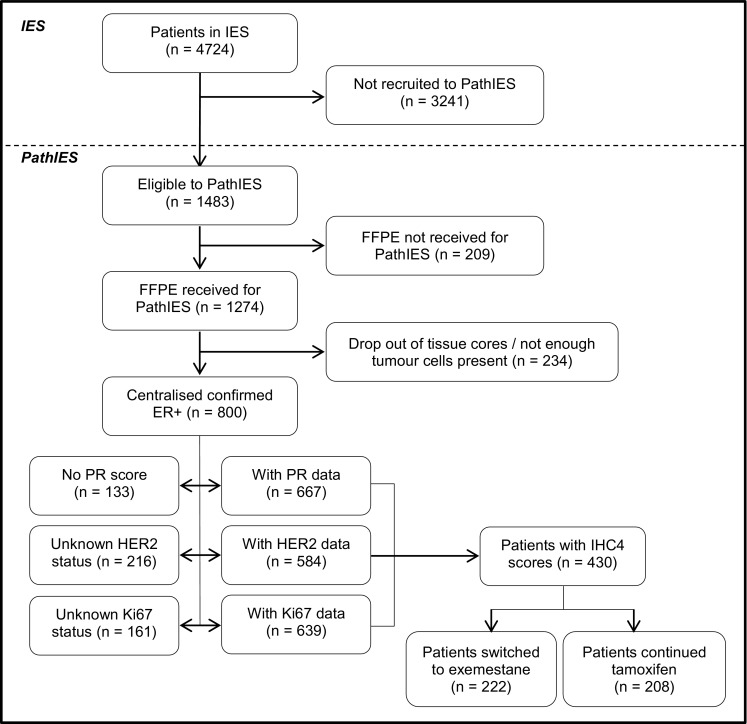
Consort diagram

Of the 1274 cases, 800 were confirmed as ER+ by centralised review (Fig. 1). Interpretable immunohistochemical data for IHC4 markers were only available for 430 women of whom 350 had complete set of data for all clinical factors used in the analysis. In these 350 patients, there were 105 recurrences of which 67 were distant recurrences. Table 1 outlines the characteristics of those patients with available data on all IHC4 markers (*N* = 430) and those without (*N* = 4294) within IES population.

**Table 1:** Comparison of patient characteristics according to data availability (N = 430 v 4294)

	Available IHC-4 markers			
	Yes (N = 430)		No (N = 4294)	
	N	%	N	%
Treatment				
Tamoxifen	208	48.4	2164	50.4
Exemestane	222	51.6	2130	49.6
Age (years)				
< 60	142	33.0	1381	32.2
60–69	191	44.4	1830	42.6
70 +	97	22.6	1083	25.2
Grade				
GI	97	22.6	692	16.1
GII	209	48.6	1778	41.4
GIII	78	18.1	845	19.7
Not assessable	2	0.5	101	2.4
Unknown	44	10.2	878	20.4
Nodes				
Negative	193	44.9	2254	52.5
1–3 N+	143	33.3	1288	30.0
>3 N+	59	13.7	599	13.9
Unavailable	35	8.1	153	3.6
Tumour size (cm)				
≤ 2	246	57.2	2539	59.1
>2 & ≤ 5	168	39.1	1548	36.1
>5	13	3.0	109	2.5
Unavailable	3	0.7	98	2.3
Histology type				
Infiltrating ductal	329	76.5	3278	76.3
Infiltrating lobular	53	12.3	609	14.2
Other	48	11.2	398	9.3
Unavailable	0	0	9	0.2
Previous chemotherapy use				
Yes	76	17.7	1466	34.1
No	354	82.3	2828	65.9
HRT use				
Yes	145	33.7	979	22.8
No	276	64.2	3211	74.8
Unavailable	9	2.1	104	2.4

### Part A: performance of IHC4 and IHC4+C on PathIES

**Fig. 2:**
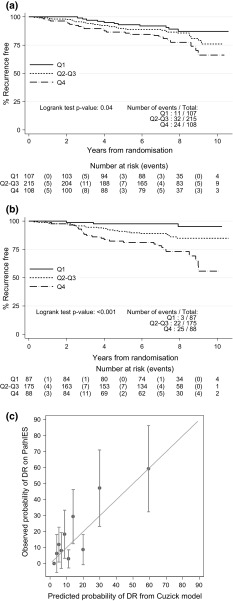
**a** Time to distant recurrence according to quartiles (Q) of IHC4 score (Q1: < 25% vs. Q2–Q3: ≥ 25% and < 75% vs. Q4: ≥ 75%) (*N* = 430) **b** Time to distant recurrence according to quartiles (Q) of the combined IHC4 + Clinical score (Q1: < 25% vs. Q2–Q3: ≥ 25% and < 75% vs. Q4: ≥ 75%) (*N* = 350). Of note, the clinical score did not include the treatment component. **c** Calibration plot of predicted versus observed probability of distant recurrence by 10 years for each 10th percentile of the IHC4 + Clinical score (*N* = 350). *DR* distant recurrence. (NB: Perfect predictions should be on the 45°line.)

Of 430 ER+ patients, 393 (91%) were PgR+, 186 (43%) expressed high proliferation (Ki67 ≥ 13%) and 20 (4.7%) were HER2+. The median IHC4 score on PathIES data was − 19.2 (IQR − 51.5, 10.2) whilst in the ATAC population this was − 4.2 (IQR − 29.9, 29.9). The HR for a change from the 1st quartile (Q1) to the Q2–Q3 was 1.45 (95% CI 0.73–2.88) and from Q1 to Q4 it was 2.32 (95% CI 1.13–4.73) (*P* = *0.04*, Fig. 2a; Table 2). Within treatment group, and possibly due to the low number of events and lack of power, IHC4 was no longer prognostic. No interaction was detected between IHC4 and treatment group (interaction *P* = *0.96*). The addition of the clinical score to the IHC4 score resulted in more profound effects in separating patients associated with differential risks (Fig. 2b; Table 2).

**Table 2:** Hazard ratio of IHC4 and IHC4 + Clinical score and IHC4 + Clinical PathIES score

Data	IHC4 score (N = 430) HR (95% CI)	IHC4 + Clinical score (N = 350) HR (95% CI)	IHC4 + Clinical PathIES score (N = 350) HR (95% CI)
PathIES			
<25%	1.00	1.00	1.00
25%-	1.45 (0.73–2.88)	3.80* (1.14–12.69)	5.54 (1.29–23.70)
75%-	2.32 (1.13–4.73)	8.96* (2.70–29.67)	15.54 (3.70–65.24)

*Unlike the Cuzick estimates these omit any treatment effect from exemestane

*HR* hazard ratio, *CI* confidence interval

Predicted probabilities of distant relapse at 10-year using IHC4+C (minus the treatment effect) were calculated. Fifty per cent of patients had predicted risk of relapse ≤ 10%, with 25% of patients with predicted risk of relapse over 20%. When comparing the observed and predicted probabilities of distant relapse at 10-year using IHC4+C (minus the treatment effect), the agreement between observed and predicted probabilities was good as shown by the calibration plot (Fig. 2c), though there is higher variability in patients with predicted risk of relapse > 10%.

### Part B: adjusting the clinical model using PathIES parameters

PathIES patients had received adjuvant tamoxifen therapy for at least 2–3 years before trial entry therefore it was of particular interest to ascertain which clinical variables remain prognostic after this time interval. Nodal status, tumour size and IHC4 demonstrated a highly significant prognostic value when associated univariately with time to distant recurrence (Table 3). Such association remained in the multivariable model after backwards selection. The modified PathIES prognostic score was calculated as

**Table 3:** Prognostic value of the IHC4 score and clinicopathological clinical factors as assessed in univariate and multivariable analysis

	Univariate				Multivariable^b^ (N = 350)		
	N	HR	95% CI (HR)	*P*	HR	95% CI (HR)	*P*
Age (year) (N = 430)							
< 65	257	1.00					
≥ 65	173	1.37	0.85–2.21	0.20			
Nodes (N = 395)							
Negative	193	1.00		< 0.001	1.00		<0.001
1–3 N+	143	1.92	1.02–3.64		1.59	0.79–3.20	
>3 N+	59	6.13	3.29–11.43		4.24	2.11–8.55	
Tumour size (cm) (N = 427)							
≤ 1	52	1.00		< 0.001			
>1– ≤ 2	194	1.90	0.56–6.38		3.93	0.52–29.55	0.03
>2– ≤3	127	3.63	1.09–12.05		5.23	0.69–39.53	
>3	54	6.47	1.91–21.88		9.11	1.19–69.91	
Tumour grade (N = 384)							
GI	97	1.00		0.46			
GII	209	1.31	0.64–2.68				
GIII	78	1.68	0.74–3.84				
IHC4^a^ (N = 430)	430	1.01	1.00–1.01	0.03	1.00	1.00–1.01	0.07
Treatment (N = 430)							
Tamoxifen	208	1.00			1.00		
Exemestane	222	0.95	0.59–1.53	0.83	0.87	0.50–1.53	0.64^b^

^a^ The IHC4 score was calculated using the Cuzick et al. algorithm and comprised ER, PR, HER2 and Ki67

^b^The multivariable model was adjusted for treatment group and was selected using a stepwise backward selection at the 10% level

**Fig. 3:**
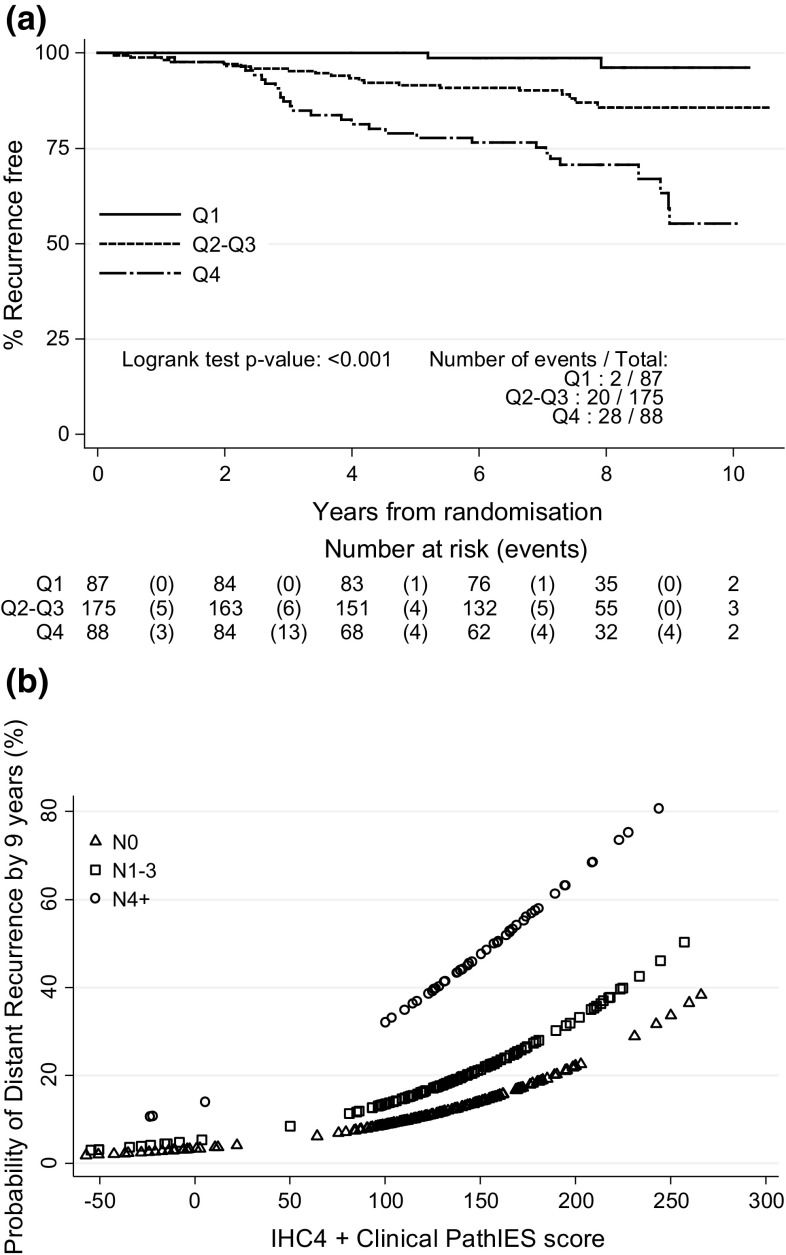
**a** Time to distant recurrence according to quartiles of the combined IHC4 + Clinical PathIES score (*N* = 350) (Q1: < 25% vs. Q2–Q3: ≥ 25% and < 75% vs. Q4: ≥ 75%) (NB: The model adjusts for the PathIES treatment effect). **b** Predicted 9-year distant recurrence probabilities for different nodal status according to IHC4 + Clinical PathIES score (*N* = 350)

${\text{IHC4}}\; + \;{\text{Clinical PathIES score}} = { 1}00x\;( - 0. 1 3x\;exe \,+ 0. 4 6 { }N_{{ 1 \text{- }3}} + 1. 4 5 { }N > _ {3} + 1. 3 7\;T_{ 1- 2} + 1. 6 5\;T_{ 1- 2} +\,{ 2}. 2 1\;T>_{3} + 0.00 4 8 {\text{ IHC4}}),$ where *N*j and *T*j denote categories of nodal status (N0, 1–3 N+, > 3 N+) and tumour size (*T*_0_, *T*_1–2_, *T*_2–3_, *T* _ >3_) as described above for the IHC4+C score, respectively. For ease of interpretation, the score has been multiplied by 100. After computing this score for all patients, and categorised into three groups with cutoffs 25% and 75%, Fig. 3a shows Kaplan–Meier curves for these groups according to the IHC4 + Clinical PathIES score.

The combined IHC4 + Clinical PathIES score was highly prognostic for outcome (*P* < *0.001)*: HR 5.54, 95% CI (1.29–23.70) for a change from Q1 to Q2–Q3 and HR 15.54, 95% CI (3.70–65.24) for a change from Q1 to Q4 (added to Table 2 for comparison). Figure 3b shows the relationship of the combined IHC4 + Clinical PathIES score with the risk of distant recurrence after 9 years according to nodal status.

## Discussion

To our knowledge, this is the first time that the IHC4 and IHC4 + Clinical score has been tested for its ability to predict relapse in a cohort of patients who switched to an AI at 2–3 years, thus excluding those who relapsed early and more closely resembling the cohort who would potentially be considered for extended adjuvant therapy beyond 5 years. We found that patients with a IHC4 + Clinical score of ≥ 75th percentile have an approximately 50% risk of recurrence by 10 years after switching at 2–3 years. This may imply that this subgroup should continue adjuvant endocrine therapy beyond the total of 5 years.

Prediction of late relapse is a matter of considerable concern for patients who have switched therapies at 2–3 years and who remain disease-free after 2–3 years, since the current and planned randomised studies are insufficiently mature to assist their decision-making at the current time.

The IHC4 score has been confirmed as being predictive of early relapse by a number of groups, and is known to be especially valuable when combined with clinical prognostic scores [10]. Recently it has been compared with other scoring systems for its ability to predict both early and late recurrences [18, 19]; although the PAM50 risk of recurrence (ROR) score was superior in this study, the IHC4 has been found to be an important scoring system.

Our results confirm the prognostic importance of IHC4, alone and in conjunction with clinical scores. Although results from the calibration plot indicated that the prediction based on the published IHC4+C derived from TransATAC study was higher than the actual observed probability in some groups of predicted risk > 10%, One possible reason for this is that PathIES patients were treated with tamoxifen for 2–3 years, and remained recurrence-free before being randomised. Our results, nevertheless, demonstrated the prognostic value of IHC4 to segregate patients associated with differential risks of recurrence. The predictive value of the calculator might be improved by adjusting the weight estimates for each of the factors, given this is a different population and potential prognostic time dependency of some of the clinical pathological variables. Additional study using an independent cohort of patients is needed to investigate the robustness of the estimates.

Several other scoring systems have been advocated for their ability to predict late recurrence in patients with ER positive breast cancer. Sestak et al. [19] compared IHC4, recurrence score (RS) as well as the PAM50 ROR score in patients enrolled in the ATAC study: here, node status, tumour size and the ROR score, a gene expression profile test, were the factors best able to predict long-term relapse. More recently, the TransATAC group compared the breast cancer index (BCI) (both linear and cubic) the OncotypeDX, as well as the IHC4 score; here the BCI (linear) had the best predictive value [20]. The components of this score that were most important were HOXB13/IL17BR. The reason for these two factors being so important appears to be that HOXB3 can over-ride the tumour suppressor p21 whilst IL17 is now known to be the prime neutrophil-dependent growth promoter in breast cancer [21]. The importance of this ratio was also underscored by the reports on retrospective analysis of the ratio in the MA17 study and predicted those who may benefit from extended letrozole therapy [22]. Recently, TransATAC group reported that EndoPredict (EPclin), an alternative test combining an eight-gene signature (EP score) with tumour size and nodal status, provided more prognostic information than the OncotypeDX score for estimating late recurrence [23], which may partly due to the reason that the test includes the significant clinicopathological variables.

Previously, an assessment of the predictive effects, in terms of therapy, of Ki67 had been reported by Viale et al. [24]. This report suggested that high Ki67 levels predicted benefit from aromatase inhibition. However, this result was not amalgamated with the other three components of the IHC4 score, namely ER, PR and HER-2. In the current study, there were too few patients to enable an assessment of the IHC4 score for its capacity to predict which patients benefit from tamoxifen or exemestane after 2–3 years.

Recently, we carried out immunohistochemical staining for ER beta 1 and 2 in a subset of patients. Here, we found that, for those patients whose tumours expressed ER beta 1, the beneficial effect of simply continuing tamoxifen was similar to the patients who switched treatment to exemestane. Although requiring confirmation, this study suggests that it may be possible to ‘tailor’ treatment according to the primary tumour characteristics. This, combined with the IHC4 + clinical score, should enable us to optimise the type and duration of endocrine therapy.

There are a few caveats before translating these results into clinical practice; firstly, these patients did not receive trastuzumab; the study was initiated before the studies of adjuvant trastuzumab were mature and adjuvant trastuzumab became standard practice for patients whose tumours expressed HER-2; however, only 5% of patients had HER2 over-expressed tumours in this study. Secondly, Ki67 measurement, despite being the subject of a recent consortium statement remains a challenging analyte in tissue sections, due principally to heterogeneity of expression [25, 26]. Thus, all the Ki67 values were analysed and assessed in one central laboratory. Secondly, a large proportion of patients received chemotherapy in this study, and especially this substudy, and caution should be exercised in translating these results to patients who did not receive cytotoxic chemotherapy.

Finally, although tissue markers reflect the biology of breast cancers in large series such as this, they do not enable clinicians to accurately predict the type and duration of treatment for individual patients; this is reflected by our finding here that approximately 50% of those with the highest quartile of the IHC4 + clinical score have not yet relapsed.

Other methods of predicting effectiveness and duration of treatment include the assessment of cell-free DNA. Using sensitive detection methods it is possible to detect circulating DNA from apoptosis residual breast cancer cells. It has now been shown that copy number variation [27] and detection of mutations [28] potentially can predict which patients are resistant to therapy.

In summary, the IHC4 score is useful in predicting long-term relapse in patients who remain disease-free after 2–3 years. Future, prospective studies are needed to define the role of IHC4 in selecting patients for long-term therapy.

## Electronic supplementary material

Below is the link to the electronic supplementary material.

Supplementary material 1 (DOCX 20 kb)
