# Supplementary material for: Evaluation of applying IHC4 as a prognostic model in the translational study of Intergroup Exemestane Study (IES): PathIES
Source: Breast Cancer Res Treat. 2017 Nov 24;168(1):169–78. doi: 10.1007/s10549-017-4543-7 (PMC5847042; doi:10.1007/s10549-017-4543-7)
Supplement: Supplementary file 1 — Supplementary material 1 (DOCX 20 kb) [file 10549_2017_4543_MOESM1_ESM.docx]

**Supplementary Table 1: Baseline clinical characteristics for patients who did and did not have IHC4 score within PathIES participating sites and in non-participating sites.**

*HRT: hormone replacement therapy. * Chi^2^ test calculation includes only Grades 1, 2, 3/undifferentiated.*

|  | Centres that provided tissue | | | | | Centres that did not provide tissue | | |
| --- | --- | --- | --- | --- | --- | --- | --- | --- |
|  | Patients with IHC4 score | Col % | Patients without tissue /score | Col % | Patients without tissue  /score | | | Col % |
| Tumour grade |  |  |  |  |  | | |  |
| 1 | 97 | 23 | 175 | 16 | 517 | | | 16 |
| 2 | 209 | 49 | 424 | 39 | 1354 | | | 42 |
| 3 / Undifferentiated | 78 | 18 | 200 | 19 | 645 | | | 20 |
| Not assessable | 2 | 0 | 25 | 2 | 76 | | | 2 |
| Unknown | 44 | 10 | 252 | 23 | 626 | | | 19 |
| *Chi^2^ trend test - within centre** | *p=0.15* | | | | |  |  | |
| *Chi^2^ trend test - with and without tissue/score** | *p=0.03* | | | | | | | |
| Treatment arm |  |  |  |  |  | |  | |
| Exemestane | 222 | 52 | 536 | 50 | 1594 | | 50 | |
| Tamoxifen | 208 | 48 | 540 | 50 | 1624 | | 50 | |
| *Chi^2^ test - within centre* | *p=0.53* | | | | |  |  | |
| *Chi^2^ test - with and without tissue/score* | *p=0.42* | | | | | | | |
| Nodal status |  |  |  |  |  | |  | |
| Negative | 193 | 49 | 483 | 48 | 1771 | | 56 | |
| 1-3 N+ | 143 | 36 | 377 | 37 | 911 | | 29 | |
| 4-9 N+ | 42 | 11 | 115 | 11 | 329 | | 10 | |
| ≥10 N+ | 17 | 4 | 40 | 4 | 115 | | 4 | |
| *Chi^2^ trend test - within centre* | *p=0.94* | | | | |  |  | |
| *Chi^2^ trend test - with and without tissue/score* | *p=0.15* | | | | | | | |
| Age group |  |  |  |  |  | | |  |
| <60 | 142 | 33 | 350 | 33 | 1031 | | | 32 |
| 60 - 69 | 191 | 44 | 481 | 45 | 1349 | | | 42 |
| 70+ | 97 | 23 | 245 | 23 | 838 | | | 26 |
| *Chi^2^ test - within centre* | *p=0.98* | | | | |  |  | |
| *Chi^2^ test - with and without tissue/score* | *p=0.47* | | | | | | | |
| Previous chemotherapy use |  |  |  |  |  | |  | |
| No | 354 | 82 | 849 | 79 | 1979 | | 62 | |
| Yes | 76 | 18 | 227 | 21 | 1239 | | 39 | |
| *Chi^2^ trend test - within centre* | *p=0.14* | | | | |  |  | |
| *Chi^2^ trend test - with and without tissue/score* | *p<0.001* | | | | | | | |
| HRT use |  |  |  |  |  | |  | |
| Yes | 145 | 34 | 289 | 28 | 690 | | 22 | |
| No | 276 | 66 | 734 | 72 | 2477 | | 78 | |
| *Chi^2^ test - within centre* | *p=0.02* | | | | |  |  | |
| *Chi^2^ test - with and without tissue/score* | *p<0.001* | | | | | | | |
| Histological type |  |  |  |  |  | |  | |
| Infiltrating ductal | 329 | 77 | 775 | 72 | 2503 | | 78 | |
| Infiltrating lobular | 53 | 12 | 172 | 16 | 437 | | 14 | |
| Other | 48 | 11 | 129 | 12 | 269 | | 8 | |
| *Chi^2^ test - within centre* | *p=0.15* | | | | |  |  | |
| *Chi^2^ test - with and without tissue/score* | *p=0.30* | | | | | | | |
| Tumour size group (cm) |  |  |  |  |  | |  | |
| ≤2cm | 246 | 58 | 640 | 61 | 1899 | | 60 | |
| >2 - ≤5cm | 168 | 39 | 377 | 36 | 1171 | | 37 | |
| >5cm | 13 | 3 | 25 | 2 | 84 | | 3 | |
| *Chi^2^ trend test - within centre* | *p=0.36* | | | | |  |  | |
| *Chi^2^ trend test - with and without tissue/score* | *p=0.48* | | | | | | | |
